# High-Fat Diet Differentially Regulates Fibroblast Growth Factor Expression in Metabolic Tissues of Young and Aged Male Mice

**DOI:** 10.1210/jendso/bvaf096

**Published:** 2025-05-29

**Authors:** Qian Lin, Xiaodan Hui, Chunjie Gu, Kyounghee Min, Lijuan Xiong, Wenqian Zhou, Jason Xu, Sara E Watson, Kupper A Wintergerst, Lu Cai, Zhongbin Deng, Yi Tan

**Affiliations:** Touchstone Diabetes Center, The University of Texas Southwestern Medical Center at Dallas, Dallas, TX 75390, USA; Pediatric Research Institute, Departments of Pediatrics, Pediatric Research Institute, University of Louisville School of Medicine, Louisville, KY 40202, USA; Pediatric Research Institute, Departments of Pediatrics, Pediatric Research Institute, University of Louisville School of Medicine, Louisville, KY 40202, USA; Pediatric Research Institute, Departments of Pediatrics, Pediatric Research Institute, University of Louisville School of Medicine, Louisville, KY 40202, USA; Touchstone Diabetes Center, The University of Texas Southwestern Medical Center at Dallas, Dallas, TX 75390, USA; Pediatric Research Institute, Departments of Pediatrics, Pediatric Research Institute, University of Louisville School of Medicine, Louisville, KY 40202, USA; Pediatric Research Institute, Departments of Pediatrics, Pediatric Research Institute, University of Louisville School of Medicine, Louisville, KY 40202, USA; Pediatric Research Institute, Departments of Pediatrics, Pediatric Research Institute, University of Louisville School of Medicine, Louisville, KY 40202, USA; Wendy Novak Diabetes Institute, Norton Children's Hospital, Louisville, KY 40202, USA; Norton Children's Endocrinology, Department of Pediatrics, University of Louisville, Norton Children's Hospital, Louisville, KY 40202, USA; Wendy Novak Diabetes Institute, Norton Children's Hospital, Louisville, KY 40202, USA; Norton Children's Endocrinology, Department of Pediatrics, University of Louisville, Norton Children's Hospital, Louisville, KY 40202, USA; The Center for Integrative Environmental Health Sciences, University of Louisville School of Medicine, Louisville, KY 40202, USA; Pediatric Research Institute, Departments of Pediatrics, Pediatric Research Institute, University of Louisville School of Medicine, Louisville, KY 40202, USA; Wendy Novak Diabetes Institute, Norton Children's Hospital, Louisville, KY 40202, USA; The Center for Integrative Environmental Health Sciences, University of Louisville School of Medicine, Louisville, KY 40202, USA; Department of Pharmacology and Toxicology, University of Louisville School of Medicine, Louisville, KY 40202, USA; Department of Radiation Oncology, University of Louisville School of Medicine, Louisville, KY 40202, USA; Department of Surgery, Division of Immunotherapy, University of Louisville School of Medicine, KY 40202, USA; Brown Cancer Center, University of Louisville School of Medicine, Louisville, KY 40202, USA; Pediatric Research Institute, Departments of Pediatrics, Pediatric Research Institute, University of Louisville School of Medicine, Louisville, KY 40202, USA; Wendy Novak Diabetes Institute, Norton Children's Hospital, Louisville, KY 40202, USA; Department of Pharmacology and Toxicology, University of Louisville School of Medicine, Louisville, KY 40202, USA

**Keywords:** fibroblast growth factors, metabolic syndromes, obesity, aging, tissue-specific dynamics

## Abstract

**Context:**

Fibroblast growth factors (FGFs) play critical roles in metabolism, yet their tissue-specific expression in response to obesity and aging remains unclear.

**Objective:**

We investigated the mRNA expression profiles of FGFs along with their receptors, across major metabolic tissues—heart, liver, kidney, skeletal muscle, gonadal white adipose tissue, subcutaneous white adipose tissue, and brown adipose tissue.

**Methods:**

Young (7-week-old) and aged (12-month-old) male mice were fed either a high-fat diet (HFD) or a normal diet for 11 weeks. Real-time quantitative polymerase chain reaction was used to measure mRNA expression levels of FGFs and their receptors.

**Results:**

The FGF system exhibited widespread expression, with the heart showing the most pronounced response to HFD-induced stress, followed by the liver and kidney, while skeletal muscle exhibited minimal changes. Adipose tissues displayed distinct FGF expression patterns under HFD conditions, with FGF1 being the most responsive, followed by FGF9. Although aged mice exhibited significantly greater body and organ weights, FGF expression profiles showed minimal variation between young and aged mice in most organs except heart.

**Conclusion:**

These findings underscore the tissue-specific dynamics of the FGF system under metabolic stress, identifying FGF1 as a promising therapeutic target for addressing obesity- and aging-related metabolic dysfunction.

The fibroblast growth factor (FGF) family comprises 22 members with diverse roles in cellular development, metabolic homeostasis, and disease regulation. FGFs are classified into paracrine (FGF1-10, FGF16-18, FGF20, and FGF22), endocrine (FGF15/FGF19, FGF21, and FGF23), and intracrine FGFs (FGF11-FGF14) [1, 2]. Most of these growth factors exert their biological functions by binding to specific tyrosine kinase receptors (FGFR1-4) and activating intracellular signaling pathways [3, 4]. The specificity of FGF–FGFR binding is tightly regulated, with receptor isoforms (eg, FGFR1b, FGFR1c, FGFR2b, and FGFR2c) providing tissue-specific signaling responses.

Paracrine FGFs, also referred to as “mitogenic FGFs,” act locally and require heparin/heparan sulfate as a cofactor for FGFR dimerization, mediating multiple developmental and physiological processes [5]. Their release is modulated by secreted FGF binding proteins (eg, FGFBP1), which fine-tune FGFR signaling [2]. Endocrine FGFs, on the other hand, rely on Klotho family coreceptors, including Klotho, β-Klotho, and Lctl (lactase like) to activate FGFRs and regulate systemic processes such as energy homeostasis and nutrient metabolism [5, 6]. In contrast, intracrine FGFs are intracellular proteins and act independently of cell surface FGFRs to primarily regulate the electrical excitability of neurons [7]. This classification underscores the highly specialized and diverse roles of FGFs in maintaining physiological balance.

Obesity is a major risk factor for metabolic syndromes, including type 2 diabetes and cardiovascular disease [8, 9]. Among FGFs, FGF21 has been extensively studied for its role as a metabolic regulator, enhancing insulin sensitivity, modulating body weight, and reducing lipid accumulation through its actions on the liver [10], adipose tissue [11, 12], and skeletal muscle [13]. Similarly, FGF15/FGF19, secreted from the intestine, regulates hepatic and bile acid metabolism, showing promise in obesity-related disorders [14, 15].

Interestingly, mitogenic FGFs such as FGF1, FGF2, FGF4, and FGF6 have recently been implicated in metabolic regulation, expanding beyond their traditional roles in wound healing [16-18], ischemic injury [19], and nerve repair [20]. For instance, FGF1 improves glucose homeostasis and lipid metabolism, making it a promising therapeutic candidate for diabetes and obesity [3, 21, 22]. FGF4 exerts antihyperglycemic effects [23], protects against hepatic steatosis in metabolic-associated fatty liver disease [24] and mediates bile acid homeostasis under cholestatic stress [25]. Additionally, FGF6 enhances whole-body metabolism by targeting skeletal muscle [26]. These findings highlight the dual roles of mitogenic FGFs in both growth promotion and metabolic regulation.

Indeed, dysregulated expression of FGFs under metabolic stress has been documented. For example, FGF1 expression increases in adipose tissue under high-fat diet (HFD) conditions, promoting adipose tissue remodeling [21]. A recent study by Kim and colleagues further expanded our understanding by showing that silencing FGF11 in the hypothalamus of mice prevents metabolic disorders in diet-induced obesity, highlighting the important role of FGF11 in regulating metabolism at the central level [27]. Similarly, hepatic FGF21 expression is elevated in metabolic-associated fatty liver disease, acting as a compensatory response to maintain metabolic balance [28, 29]. These findings underscore the therapeutic potential of FGFs in addressing obesity and its complications.

Age exacerbates metabolic dysfunction, with obesity further contributing to epigenetic changes that drive insulin resistance and metabolic syndrome [30]. Despite growing recognition of these processes, the roles of FGFs and their coreceptors in aging, particularly in metabolic disorders, remain poorly understood. Notably, the antiaging hormone Klotho has been shown to protect against renal insufficiency [31] and heart failure [32]. However, the broader roles of FGFs and their receptors in aging-associated metabolic dysfunction require further exploration.

In this study, we investigated the effects of HFD-induced metabolic stress on the mRNA expression pattern of the FGF family in young (7 weeks old) and aged (12 months old) male mice. We comprehensively profiled the expression of 22 FGF family members and 10 coreceptors across key metabolic organs using real-time quantitative polymerase chain reaction (qPCR). By characterizing the tissue-specific dynamics of the FGF family under obesity and aging conditions, this work aims to uncover new therapeutic targets for metabolic disorders, with particular emphasis on the roles of paracrine FGFs that have been less extensively studied in this context.

## Materials and Methods

### Animal Models

Male C57BL/6J mice (Stock No: 000664) were obtained from Jackson Laboratory (Bar Harbor, ME) and were housed under a controlled 12:12-hour light/dark cycle and maintained at constant temperature conditions. Food and water were provided ad libitum. All experimental procedures were approved by the Institutional Animal Care and Use Committee of the University of Louisville.

### HFD Feeding in Young and Aged Mice

Young mice (7 weeks old) and aged mice (12 months old) were randomly assigned to either a HFD (TD.09766, 60% kcal from fat, Envigo, Madison, WI) or a normal diet (ND) (TD120455, 10% kcal from fat, Envigo, Madison, WI) for 11 weeks, forming 4 experimental groups: ND-Young (n = 7), HFD-Young (n = 8), ND-Aged (n = 6), and HFD-Aged (n = 7). Fasting blood glucose levels and body weight were recorded at designated time points, with blood glucose levels measured using a FreeStyle complete blood glucose monitor (Abbott Diabetes Care Inc., Alameda, CA). Upon study completion, mice were humanely euthanized, and tissues including the heart, liver, kidney, skeletal muscle, gonadal white adipose tissue (gWAT), brown adipose tissue (BAT) and subcutaneous white adipose tissue (sWAT) were collected for further analysis. Tissue weights were normalized to tibial length.

### Intraperitoneal Glucose Tolerance Test (IPGTT) and Intraperitoneal Insulin Tolerance Test (IPITT)

The IPGTT [22] and IPITT [33] were performed after a 6-hour fast. Mice were injected intraperitoneally with glucose solution (1 g/kg) and Humulin R (0.75 U/kg), respectively, and blood glucose levels were measured at 0, 15, 30, 60, 90, and 120 minutes postinjection. The blood glucose levels over time were plotted, and the area under the curve was calculated to quantify glucose tolerance or insulin sensitivity.

### Plasma Insulin Measurement

Plasma insulin levels were quantified using the Ultra-Sensitive Mouse Insulin ELISA Kit (Catalog: 90080, Crystal Chem, IL, USA) according to the manufacturer's instruction.

### Fat Composition Analysis

Body fat composition was assessed using a Lunar PIXImus Dual-Energy X-Ray Absorptiometer Systems (Micro Photonics Inc., Allentown, PA).

### RNA Preparation, cDNA Analysis, and Real-Time qPCR

Total RNA was extracted from tissues using TRIzol reagent (Invitrogen, Carlsbad, CA). RNA concentration was determined with a Nanodrop ND-1000 spectrophotometer. For each sample, 1 µg of RNA was reverse transcribed into cDNA using a reverse transcription kit (Promega, Madison, WI). RT-qPCR was performed in duplicate on a LightCycler 96 system (Roche, Indianapolis, IN) using Power SYBR Green mix, with 18s rRNA as the housekeeping gene. Primers were designed and synthesized by Thermo Fisher Scientific Inc. (Grand Island, NY) based on previously published sequences [34]. Details are provided in Tables 1 and 2.

**Table 1.** Primers of real-time PCR for fibroblast growth factors (FGFs)

Genes	Primer sequence
FGF1	5′-ACACCGAAGGGCTTTTATACG-3′ 5′-GTGTAAGTGTTATAATGGTTTTCTTCCA-3′
FGF2	5′-CAACCGGTACCTTGCTATGA-3′ 5′-TCCGTGACCGGTAAGTATTG-3′
FGF3	5′-ACGGCAGCCTTGAGAACA-3′ 5′-CCACTTCCACCGCAGTAATC-3′
FGF4	5′-CGGCTCTACTGCAACGTG-3′ 5′-CGGAGAGAGCTCCAGAAGAC-3′
FGF5	5′-CTGCAGATCTACCCGGATG-3′ 5′TCCTCGTATTCCTACAATCCC-3′
FGF6	5′-CTGTACACAACGCCCAGCTT-3′ 5′-TTGTTTGGAAGGAGGGTTTCTC-3′
FGF7	5′-AAGGGACCCAGGAGATGAAG-3′ 5′-ACTGCCACGGTCCTGATTT-3′
FGF8	5′-CATGGCAGAAGACGGAGAC-3′ 5′-ACTCGGACTCTGCTTCCAAA-3′
FGF9	5′-CTATCCAGGGAACCAGGAAAGA-3′ 5′-CAGGCCCACTGCTATACTGATAAA-3′
FGF10	5′-GCGGGACCAAGAATGAAGA-3′ 5′-AGTTGCTGTTGATGGCTTTGA-3′
FGF11	5′-TTGTACAGCTCGCCACATTTC-3′ 5′-GTAATTCTCAAAGACGCACTCCTT-3′
FGF12	5′-CATTTTGTACCAAAACCTATTGAAGTG-3′ 5′-TCCTTGAGCGTCCTTGCTT-3′
FGF13	5′-AATGAACAGCGAGGGATACTTG-3′ 5′-ACTGATTCTTTGAATTTGCACTCA-3′
FGF14	5′-CCCGATGGAGCTCTCGAT-3′ 5′-GGTTGAACAGTGTGGAATTGGT-3′
Fgf15	5′-ACGGGCTGATTCGCTACTC-3′ 5′-TGTAGCCTAAACAGTCCATTTCCT-3′
FGF16	5′-GGCCTGTACCTAGGAATGAATGA-3′ 5′-TTCCCGGAAAACACATTCAC-3′
FGF17	5′-GGCAAATCCGTGAATACCA-3′ 5′-CTGCTGCCGAATGTATCTGT-3′
FGF18	5′-TGCTGTGCTTCCAGGTTCA-3′ 5′-GGATGCGGAAGTCCACATT-3′
FGF20	5′-CGGCAGGATCACAGTCTCTT-3′ 5′-CCAGTCCCACTGCCACACT-3′
FGF21	5′-CCTCTAGGTTTCTTTGCCAACAG-3′ 5′-AAGCTGCAGGCCTCAGGAT-3′
FGF22	5′-GTGGGCACTGTGGTGATCA-3′ 5′-GCGATTCATGGCCACATAGA-3′
FGF23	5′-CCCCCATCAGACCATCTACA-3′ 5′-TTCGAGTCATGGCTCCTGTT-3′

**Table 2.** Primers of real-time PCR for fibroblast growth factor receptors (FGFRs)

Genes	Primer sequence
FGFR1b	5′-CAACTTGCCGTATGTCCAGATC-3′ 5′-CTCCGCATCCGAGCTATTAA-3′
FGFR1c	5′-GCCAGACAACTTGCCGTATG-3′ 5′-ATTTCCTTGTCGGTGGTATTAACTC-3′
FGFR2b	5′-GGGCTGCCCTACCTCAAG-3′ 5′-CTTCTGCATTGGAGCTATTTATCC-3′
FGFR2c	5′-CCCGGCCCTCCTTCA-3′ 5′-GTTGGGAGATTTGGTATTTGGTT-3′
FGFR-3b	5′-GCACGCCCTACGTCACTGTA-3′ 5′-GCGTCTGCCTCCACATTCT-3′
FGFR-3c	5′-ACGGCACGCCCTACGT-3′ 5′-CTCCTTGTCGGTGGTGTTAGC-3′
FGFR4	5′-CGCCAGCCTGTCACTATACAAA-3′ 5′-CCAGAGGACCTCGACTCCAA-3′
Klotho	5′-AATTATGTGAATGAGGCTCTGAAAG-3′ 5′-TACGCAAAGTAGCCACAAAGG-3′
Klotho-β	5′-GATGAAGAATTTCCTAAACCAGGTT-3′ 5′-AACCAAACACGCGGATTTC-3′
FGFBP1	5′-CCCCAGTACACCTGGATCTG-3′ 5′-CAGGATGAGGCTGTGGAGTCT-3′
Lctl	5′-CTTGGAAACTTGCTCCATCAAC-3′ 5′-CACGTGCATGTGCCTAAGC-3′

### Statistical Analysis

All data were expressed as mean ± SEM. When there were 2 factors and multiple groups involved, F-tests for 2-way analysis of variance were used to determine whether there was a group difference. If there was a group difference, post hoc unpaired 2-tail Student t-tests were applied to examine which groups were significantly different. Differences were considered statistically significant when *P* < .05. Only the expression levels of FGFs, FGFRs, or coreceptors that were fully detectable in the target organ or tissue samples were statistically analyzed.

## Results

### Metabolic Disorders Induced by HFD in Young and Aged Mice

**Figure 1.**
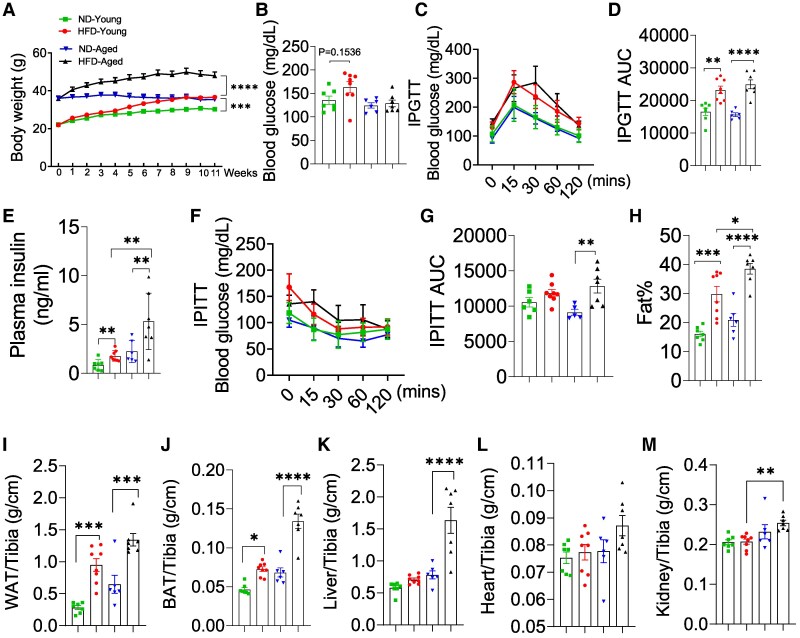
Characterization of metabolic differences between young and aged mice fed a HFD. Young (7 weeks old) and aged mice (12 months old) were fed HFD (HFD-Young (n = 8) and HFD-Aged (n = 7)) for 11 weeks. Normal diet (ND) controls included ND-Young (n = 7) and ND-Aged (n = 6). (A) Body weight; (B) blood glucose; (C, D) intraperitoneal glucose tolerance test (IPGTT) and area under the curve (AUC) analysis; (E) plasma insulin level; (F, G) intraperitoneal insulin tolerance test (IPITT) and AUC analysis; (H) fat content as percentage of body weight; organs weights relative to tibial length: (I) heart; (J) liver; (K) WAT; (L) BAT; (M) kidney. Data are mean ± SEM. *P < .05; **P < .01; ***P < .001, and ****P < .0001.

HFD-fed mice exhibited significant weight gain (Fig. 1A) and elevated fasting glucose levels (Fig. 1B) compared with ND-fed controls. IPGTT (Fig. 1C and 1D), plasma insulin levels (Fig. 1E) and IPITT results (Fig. 1G and 1H) indicated impaired glucose tolerance and insulin resistance in HFD-fed groups, with aged mice showing more pronounced metabolic dysfunction. HFD-fed mice also showed substantial increases in fat mass (Fig. 1H), along with elevated weights of gWAT (Fig. 1I) and BAT (Fig. 1J). Liver weight significantly increased in HFD-Aged mice but remained unchanged in HFD-Young mice compared with ND controls (Fig. 1K). Heart (Fig. 1L) and kidney (Fig. 1M) weights were largely unaffected in young mice, while kidney weight was notably higher in HFD-Aged mice than in HFD-Young mice.

### mRNA Expression Profiles of FGFs and FGFRs in Heart

**Figure 2.**
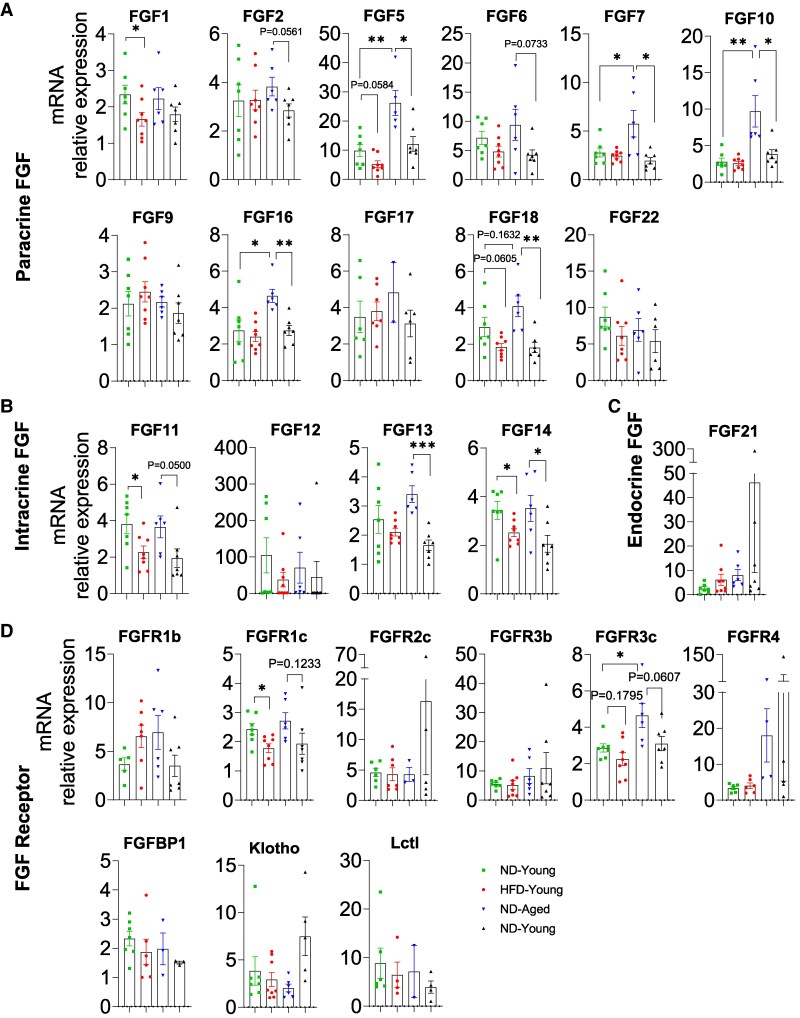
Comprehensive mRNA expression profile of the FGF family in the heart of HFD-fed young and aged mice. Expressions of (A) paracrine FGFs (FGF1, 2, 5, 6, 7, 9, 10, 16, 17, 18, and 22), (B) intracrine FGFs (FGF11, 12, 13, and 14), (C) endocrine FGF21, and (D) FGFRs (FGFR1b, R1c, R2c, R3b, R3c, R4, FGFBP1, Klotho and Lctl) in heart. Data are mean ± SEM. *P < .05; **P < .01 and ***P < .001.

Of the 15 paracrine FGFs, 11 members were fully detectable in the heart (Fig. 2A). Notably, FGF5, FGF7, FGF10, FGF16, and FGF18 showed higher expression in ND-Aged mice than in ND-Young mice, indicating an age-dependent physiological regulation (Fig. 2A). In HFD-Young mice, FGF1, FGF5, and FGF18 were significantly or marginally reduced compared with ND-Young mice. While in HFD-Aged mice, most paracrine FGFs, including FGF2, FGF5, FGF6, FGF7, FGF10, FGF16, and FGF18, were significantly or marginally reduced compared with ND-Aged mice (Fig. 2A).

Intracrine FGFs (FGF11-FGF14) were abundantly expressed in the heart (Fig. 2B). HFD-fed mice exhibited reduced levels of FGF11 and FGF14 compared with ND controls in both young and aged mice except FGF13, which was only observed in HFD-Aged mice. Only FGF21 among endocrine FGFs was expressed in the heart, with its levels unaffected by diet or age (Fig. 2C).

FGFR1b, FGFR1c, FGFR3b, FGFR3c, and Klotho were detectable in the hearts (Fig. 2D). FGFR3c was upregulated in ND-Aged hearts compared with ND-Young hearts. Notably, HFD feeding downregulated both FGFR1c and FGF3c isoform expression in HFD-Young and HFD-Aged hearts. FGFR1b, FGFR3b, and Klotho expression remained unchanged in all groups of mice regardless of age or diet (Fig. 2D).

### mRNA Expression Profiles of FGFs and FGFRs in Liver

**Figure 3.**
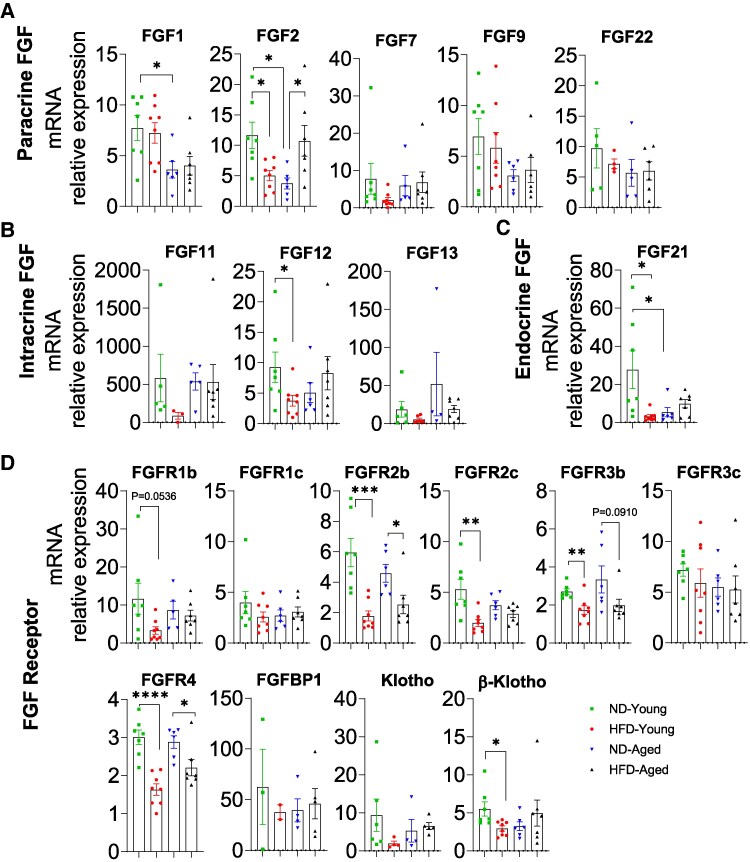
Comprehensive mRNA expression profile of the FGF family in the Liver of HFD-fed young and aged mice. Expressions of (A) paracrine FGFs (FGF1, 2, 7, 9, 18, and 22), (B) intracrine FGFs (FGF11, 12, and 13), (C) endocrine FGFs (FGF21 and 23), and (D) FGFRs (FGFR1b, R1c, R2b, R2c, R3b, R3c, R4, FGFBP1, Klotho, and β-Klotho) in liver. Data are mean ± SEM. *P < .05, **P < .01, ***P < .001, and ****P < .0001.

The liver expressed a limited subset of paracrine FGFs (FGF1, FGF2, FGF7, FGF9, and FGF22), with limited changes observed (Fig. 3A). Both FGF1 and FGF2 levels were lower in ND-Aged mice than in ND-Young mice, while FGF2 expression was decreased in HFD-Young mice but increased in HFD-Aged mice compared with ND-fed controls.

Of intracrine FGFs, only FGF12 mRNAs were fully detectable in the liver and significantly reduced in HFD-Young mice compared with ND-Young mice (Fig. 3B). For endocrine FGF21 was abundantly expressed but significantly decreased in HFD-Young mice and ND-Aged liver (Fig. 3C).

Most FGFR isoforms and coreceptors were expressed in the liver, with significant alterations in response to HFD. In young mice, HFD feeding significantly reduced the expression of FGFR1b, FGFR2b, FGFR2c, FGFR3b, FGFR4, and β-Klotho (Fig. 3D). However, in aged mice, only FGFR2b and FGFR4 showed significant reductions in response to HFD. No significant differences were observed for FGFR1c, FGFR3c, and Klotho across any experimental groups (Fig. 3D).

### mRNA Expression Profiles of FGFs and FGFRs in Kidney

**Figure 4.**
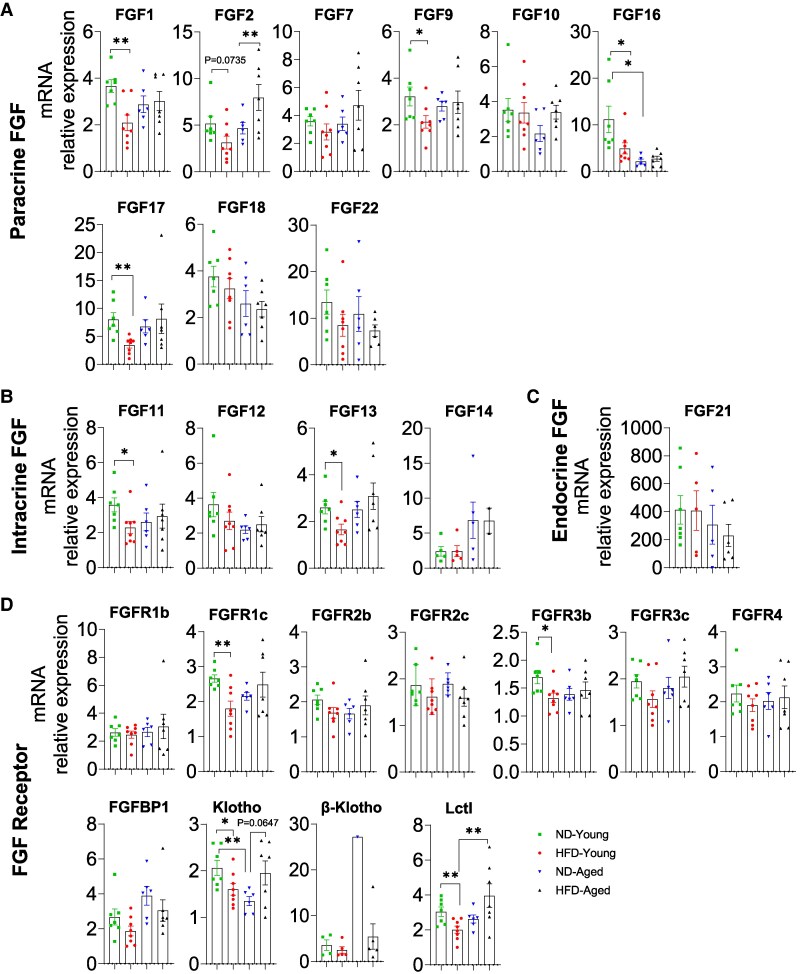
Comprehensive mRNA expression profile of the FGF family in the Kidney of HFD-fed young and aged mice. Expressions of (A) paracrine FGFs (FGF1, 2, 7, 9, 10, 16, 17, 18, and 22), (B) intracrine FGFs (FGF11, 12, 13, and 14), (C) endocrine FGF21, and (D) FGFRs (FGFR1b, R1c, R2b, R2c, R3b, R3c, R4, FGFBP1, Klotho, β-Klotho, and Lctl) in kidney. Data are mean ± SEM. *P < .05 and **P < .01.

HFD feeding significantly altered FGF expression in the kidney. Paracrine FGF1, FGF2, FGF9, FGF16, and FGF17 were downregulated in HFD-Young mice compared with ND-Young mice, while FGF2 levels were markedly increased in HFD-Aged mice and FGF16 levels were markedly decreased in ND-Aged mice (Fig. 4A). However, there were no significant differences observed for FGF7, FGF10, FGF18, and FGF22 among the groups of mice regardless of diet or age (Fig. 4A). Intracrine FGF11, FGF12, and FGF13 were all detectable in the kidney, with FGF11 and FGF13 significantly reduced in HFD-Young mice compared with ND-Young mice, while FGF14 was not consistently detectable across all samples (Fig. 4B). Endocrine FGF21 was abundantly expressed in the kidneys, but its expression did not significantly differ among experimental groups (Fig. 4C).

Except β-Klotho, all FGFRs and coreceptors were fully detectable in kidney samples, with FGFR1c, FGFR3b, Klotho, and Lctl downregulated in HFD-Young mice. Notably, both Klotho and Lctl were also downregulated in ND-Aged mice but upregulated by HFD feeding. However, there were no significant differences observed for FGFR1b, FGFR2b, FGFR2c, FGFR3c, FGFR4, and FGFBP1 among the groups of mice regardless of diet or age (Fig. 4D).

### mRNA Expression Profiles of FGFs and FGFRs in Skeletal Muscle

**Figure 5.**
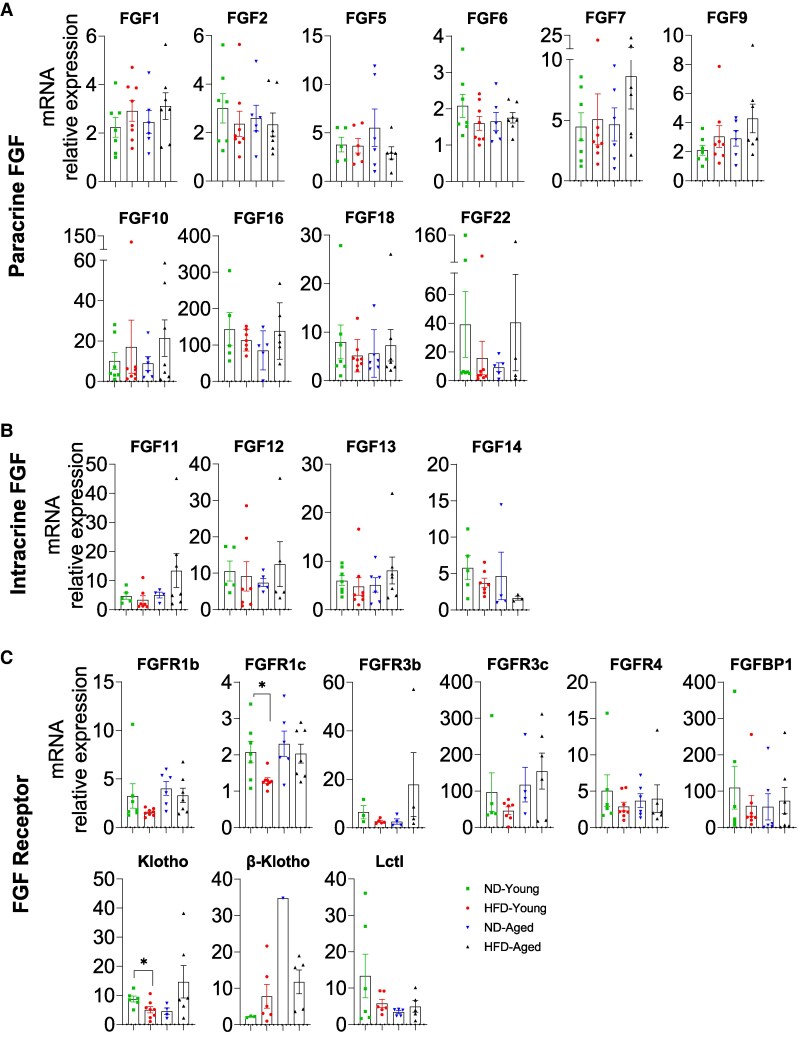
Comprehensive mRNA expression profile of the FGF family in skeletal muscle of HFD-fed young and aged mice. Expressions of (A) paracrine FGFs (FGF1, 2, 5, 6, 7, 9, 10, 16, 18, and 22), (B) intracrine FGFs (FGF11, 12, 13, and 14), and (C) FGFRs (FGFR1b, R1c, R3b, R3c, R4, FGFBP1, Klotho, β-Klotho, and Lctl) in skeletal muscle. Data are mean ± SEM. *P < .05.

Skeletal muscle showed the least responsiveness to age and diet among all tissues examined, with most FGF family members maintaining stable expression across experimental conditions. Most paracrine and intracrine FGFs were abundantly expressed in skeletal muscle, but their expression levels remained largely unchanged regardless of age or diet (Fig. 5A and 5B).

Among FGFRs and coreceptors, only FGFR1c and Klotho levels were significantly decreased in HFD-Young mice compared with ND-Young mice (Fig. 5C), potentially influencing skeletal muscle insulin sensitivity. However, FGFR2b, FGFR2c, and β-Klotho were not detectable in some or all samples and neither diet nor ageing had any effects on the expression levels of FGFR1b, FGFR3b, FGFR3c, FGFR4, FGFBP1, and Lctl (Fig. 5C).

### mRNA Expression Profiles of FGFs and FGFRs in gWAT

**Figure 6.**
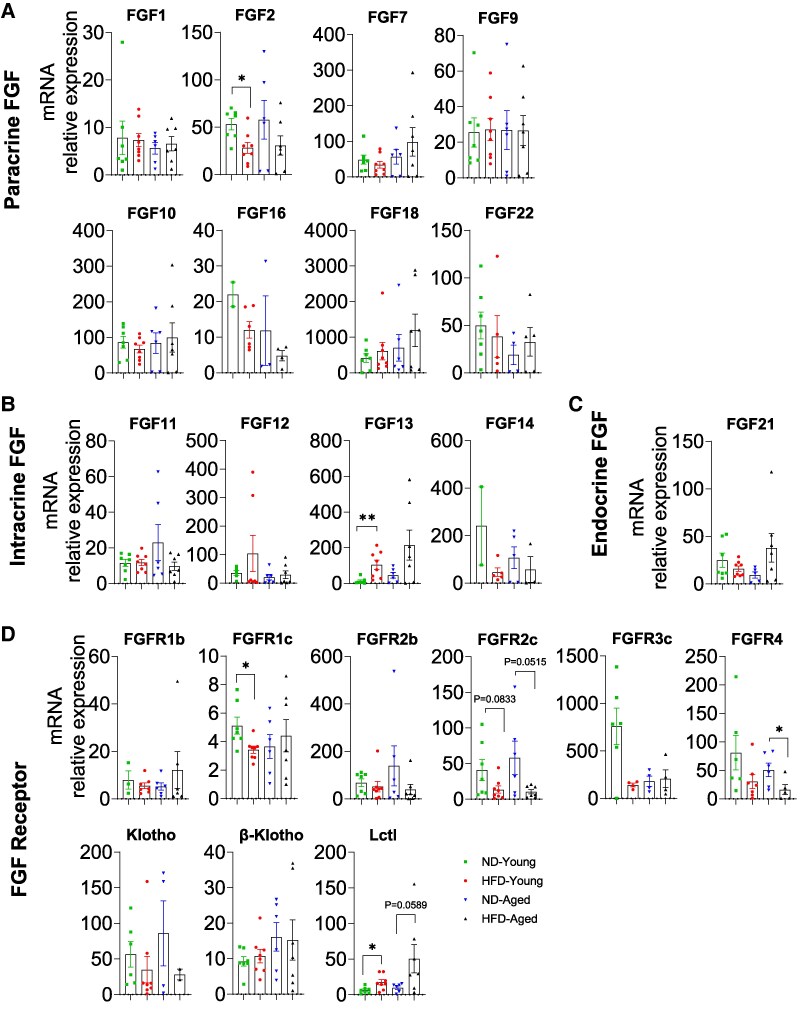
Comprehensive mRNA expression profile of the FGF family in gonadal white adipose tissue (gWAT) of HFD-fed young and aged mice. Expressions of (A) paracrine FGFs (FGF1, 2, 7, 9, 10, 16, 18, and 22), (B) intracrine FGFs (FGF11, 12, 13, and 14), (C) endocrine FGF21, and (D) FGFRs (FGFR1b, R1c, R2b, R2c, R3c, R4, FGFBP1, Klotho, β-Klotho, and Lctl) in gWAT. Data are mean ± SEM. *P < .05 and **P < .01.

Paracrine FGF2 levels in gWAT were significantly reduced in HFD-Young mice (Fig. 6A). Other paracrine FGFs, including FGF1, FGF7, FGF9, FGF10, FGF18, and FGF22, showed no significant changes across experimental groups, while the remaining paracrine FGFs were undetectable in some or all samples (Fig. 6A). Except FGF14, all intracrine FGFs were fully detectable in gWAT, but only FGF13 was increased in HFD-Young mice and the remaining intracrine FGFs remained unchanged among the groups regardless of diet and age (Fig. 6B). Endocrine FGF21 was abundantly expressed in gWAT, but its expression did not vary significantly (Fig. 6C). FGFR1c and FGFR2c were markedly downregulated in HFD-Young mice compared with ND-Young mice, and FGFR2c and FGFR4 were markedly downregulated in HFD-Aged mice compared with ND-Aged mice. In contrast, Lctl was obviously upregulated in both HFD-Young and HFD-Aged mice (Fig. 6D). However, FGFR1b, FGFR2b, FGFR3c, and β-Klotho remained unchanged among the groups of mice regardless of diet and age, and the remaining FGFRs and coreceptors were undetectable in some or all samples (Fig. 6D).

### mRNA Expression Profiles of FGFs and FGFRs in sWAT

**Figure 7.**
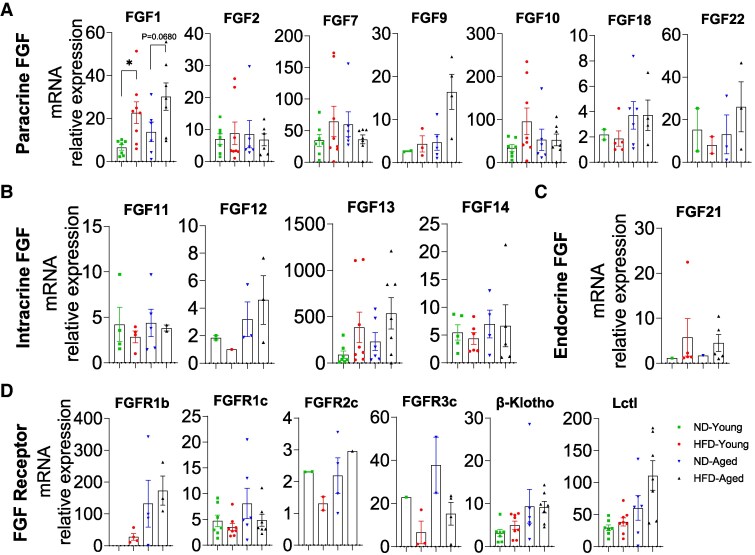
Comprehensive expression profile of the FGF family on subcutaneous white adipose tissue (sWAT) of HFD-fed young and aged mice. Expressions of (A) paracrine FGFs (FGF1, 2, 7, 9, 10, 18, and 22), (B) intracrine FGFs (FGF11, 12, 13, and 14), (C) endocrine FGF21, and (D) FGFRs (FGFR1b, R1c, R2c, R3c, β-Klotho, and Lctl) in sWAT. Data are mean ± SEM. *P < .05.

In sWAT, paracrine FGFs FGF1, FGF2, FGF7, and FGF10 were abundantly expressed, but only FGF1 showed significant upregulation in HFD-Young and HFD-Aged mice compared with their respective ND controls, whereas the remaining paracrine FGFs were undetectable in some or all samples (Fig. 7A). Among intracrine FGFs, only FGF13 was fully detectable but no differences were observed across diet and age groups (Fig. 7B). Endocrine FGF21 was inconsistently detectable across samples, while FGF15 and FGF23 were undetectable (Fig. 7C). Among FGFRs and coreceptors, only FGFR1c, β-Klotho, and Lctl were fully detectable, but their expression remained unchanged across experimental groups **(**Fig. 7D).

### mRNA Expression Profiles of FGFs and FGFRs in BAT

**Figure 8.**
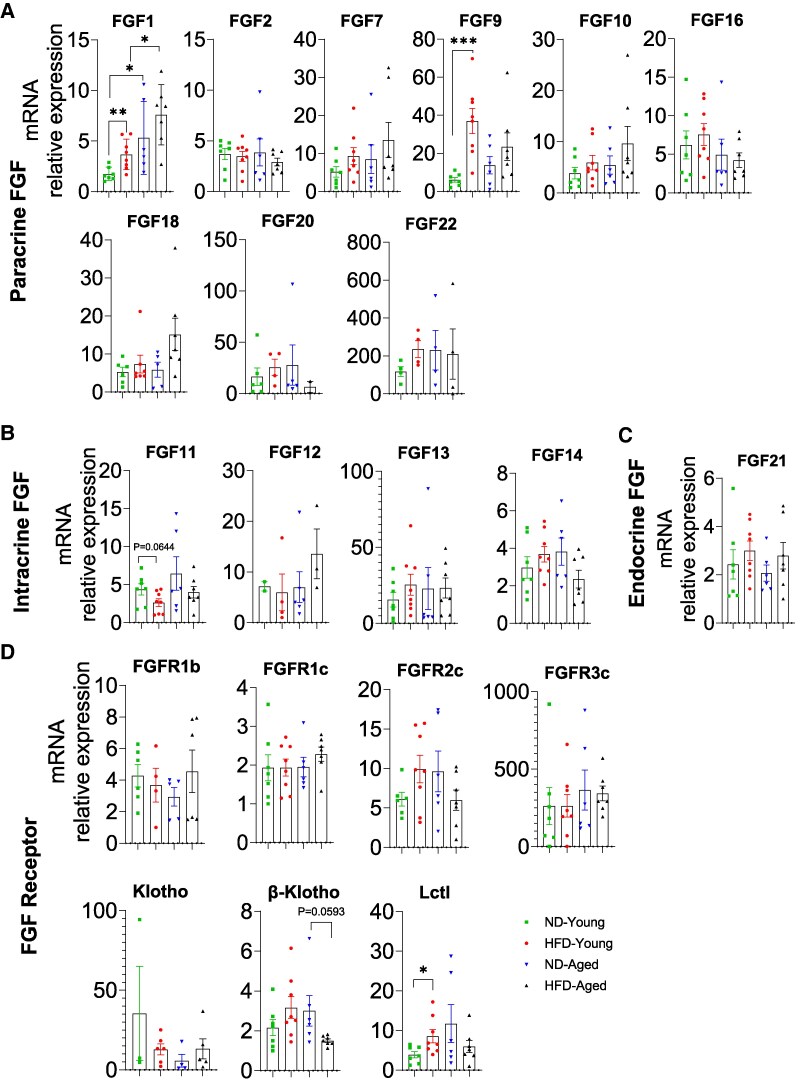
Comprehensive mRNA expression profile of the FGF family in brown adipose tissue (BAT) of HFD-fed young and aged mice. Expressions of (A) paracrine FGFs (FGF1, 2, 7, 9, 10, 16, 18, 20, and 22), (B) intracrine FGFs (FGF11, 12, 13, and 14), (C) endocrine FGF21, and (D) FGFRs (FGFR1b, R1c, R2c, R3c, Klotho, β-Klotho, and Lctl) in BAT. Data are mean ± SEM. *P < .05, **P < .01, and ***P < .001.

Paracrine FGF1, FGF2, FGF7, FGF9, FGF10, FGF16, and FGF18 were abundantly expressed in BAT, with FGF1 and FGF9 expression obviously upregulated in HFD-Young mice compared with ND-Young mice. Additionally, FGF1 expression was increased in ND-Aged mice compared with ND-Young mice, potentially indicating a role in BAT aging (Fig. 8A). The remaining paracrine FGFs were undetectable in some or all samples. Most intracrine FGFs, including FGF11, FGF13, and FGF14, were fully detectable in BAT, but only FGF11 expressions were marginally downregulated in HFD-Young mice compared with ND-Young mice (Fig. 8B). Endocrine FGF21 expression remained unchanged in all groups, while FGF15 and FGF23 were undetectable (Fig. 8C). Most FGFRs and coreceptors, including FGFR1b, FGFR1c, FGFR2c, FGFR3c, β-Klotho, and Lctl, were fully detectable in BAT, but only β-Klotho was downregulated in HFD-Aged mice compared with ND-Aged mice, whereas Lctl was upregulated in HFD-Young mice compared with ND-Young mice. The remaining FGFRs and coreceptors were undetectable in some or all samples (Fig. 8D).

## Discussion

In this study, we profiled the expression of FGFs and FGFRs across major metabolic tissues in young and aged male mice under HFD-induced metabolic stress conditions. The cardiac tissue showed the most pronounced responses, followed by liver and kidney, while skeletal muscle exhibited minimal changes. This tissue-specific sensitivity to metabolic stress may reflect the differential vulnerability of these organs to obesity- and aging-related dysfunction. Our findings highlight tissue-specific dynamics of the FGF system, providing insights into potential therapeutic targets for obesity- and aging-related metabolic disorders.

Our analysis of cardiac tissues revealed notable alterations in paracrine FGF expression under metabolic stress and aging conditions. Overall, metabolic stress had minimal effects on young mice, with only FGF1, FGF5, and FGF18 mRNA obviously decreased, whereas mRNA expression of FGF5, FGF7, FGF10, FGF16, and FGF18 was markedly upregulated with aging but downregulated with HFD feeding, indicating a higher susceptibility to metabolic stress under aging conditions. In line with our findings in FGF1 expression, a recent study demonstrated that obesity- and diabetes-induced cardiac dysfunction was accompanied by downregulation of FGF1 expression in the heart, and supplementation of exogenous FGF1 protein could sufficiently prevent obesity- and diabetes-related cardiac dysfunction and damages [35], indicating that cardiac endogenous FGF1 is essential for maintaining myocardial function under metabolic stress conditions. The pathophysiological roles of FGF5, FGF7, FGF10, FGF16, and FGF18 in hearts have been preliminarily explored. A previous study implied that FGF5 overexpression, not only in vivo in heart but also in vitro in cardiomyocytes, reduced the levels of oxidative stress and pyroptosis resulted from lipopolysaccharide [36]. FGF7 and FGF10, both part of the FGF7 subfamily [37], have been proven to be involved in cardiomyocyte survival and cardiac fibroblast activation and extracellular matrix remodeling [38-40]. FGF16, the only FGF family member that shows preferential expression in the postnatal heart [41], has been shown a direct cardioprotective effect against myocardial infarction through intramyocardial injection of FGF16 protein in *db/db* type 2 diabetic mice [42]. Similarly, FGF18 has been shown to protect against pressure overload-induced pathological cardiac hypertrophy, cardiomyocyte death, fibrosis, and dysfunction [43]. In our study, the upregulation of cardiac FGF5, FGF7, FGF10, FGF16, and FGF18 in aged mice suggests their critical roles in aging-associated cardiac adoptive responses, whereas their reduction in aged-mice with HFD feeding indicates a disruption of this adoptive mechanism, potentially accelerating metabolic syndrome-induced cardiac damage and remodeling. However, the exact roles and mechanisms of these paracrine FGFs in cardiac aging, especially under conditions of metabolic stress, need to be addressed in future studies.

Intracrine FGFs (FGF11-FGF14) play essential roles in ion channel regulation and cardiac excitability [44, 45]. It has been observed that cardiomyocyte-specific FGF13 knockout in mice led to prolonged action potential and increased arrhythmic risk [46]. The HFD-induced reduction in FGF11, FGF13, and FGF14 expression, particularly in aged mice, suggests that metabolic stress may impair cardiac conduction and increase arrhythmia susceptibility during aging.

FGF21, an endocrine FGF known for its cardioprotective effects [47], was not significantly altered by aging or HFD in hearts. However, previous studies have demonstrated that both systemic administration of recombinant FGF21 protein [48, 49] and cardiomyocyte-specific overexpression of the FGF21 gene [50] mitigate cardiac inflammation and fibrosis. The lack of significant changes in FGF21 expression suggests that cardiac endogenous FGF21 is not responsive to aging and/or metabolic stress and FGF21 mainly achieves its cardioprotective function through an endocrine mechanism.

FGFR1 and FGFR2 have been proven to mediate FGFs' cardioprotective signaling against ischemia/reperfusion injury [51]. The decline in both FGFR1c and FGFR3c expression in HFD-fed young and aged heart suggests these 2 FGFR isoforms are key mediators of cardioprotective signaling in response to metabolic stress. The upregulation of cardiac FGFR3c in aged mice implies its critical roles in mediating cardiac adoption to aging, which needs to be validated in future studies. These findings suggest that targeting specific FGFs (eg, FGF1, FGF5, FGF7, FGF10, FGF16, FGF18, and FGF21) or their receptors (FGFR1c and FGFR3c) may provide novel therapeutic strategies to combat obesity- and aging-related cardiac dysfunction.

FGF1 has been shown to mitigate diabetes-induced hepatic steatosis, fibrosis, and apoptosis, playing a crucial role in hepatic glucose and lipid homeostasis [22, 52, 53]. Herein, FGF1 exhibited age-dependent decline in expression, suggesting reduced hepatic metabolic adaptability with aging. Unlike FGF1, FGF2 expression under HFD conditions displayed age-dependent divergence, where its expression decreased in young HFD-fed mice but increased in aged HFD-fed mice. This suggests that young livers are more susceptible to HFD-induced suppression of FGF2, while aged livers may attempt to compensate by upregulating FGF2 in response to prolonged metabolic stress. However, excessive FGF2 signaling has been linked to hepatic fibrosis [54], suggesting that its upregulation in aged HFD-fed mice likely contribute to liver damage rather than protection.

Contrary to prior studies showing HFD-induced a compensatory elevation of hepatic FGF21 [55, 56], our study found that hepatic FGF21 expression declined with both aging and prolonged HFD feeding. This discrepancy might be attributable to differences in duration and composition of diet intervention [55-57]. Our findings indicate that sustained and chronic HFD-induced stress, especially in aged animals, could ultimately disrupt the compensatory transcriptional mechanisms regulating FGF21, reducing signaling efficiency and potentially accelerating obesity- and aging-associated fatty liver progression. Consistent with our observations, FGF21-knockout mice with HFD consumption develop excess fatty liver within 16 weeks [58] and with aging of 36 to 40 weeks manifest abnormal accumulation of lipids in liver [59]. Conversely, liver-specific FGF21 overexpression improves lipid metabolism and reduces hepatic inflammation [60], reinforcing the role of FGF21 in hepatic lipid homeostasis and metabolic resilience. These findings underscore its therapeutic effects in liver metabolic disorders. Indeed, there are several FGF21 analogs considered as promising options for metabolic dysfunction–associated steatohepatitis treatment, with efficacy comparable to the only Food and Drug Administration–approved medication, resmetirom [61].

In parallel with FGF expression changes, hepatic FGFR2b and FGFR4 were significantly downregulated in both young and aged HFD-fed mice. FGFR4 is a key regulator of bile acid homeostasis. Liver-specific FGFR4 knockdown in mice under HFD conditions increases bile acid synthesis and improves hepatic steatosis [62]. Its suppression in our study may indicate impaired bile acid regulation, potentially contributing to lipid accumulation and cholestatic liver disease. Similarly, FGFR2b plays a role in hepatocyte survival and liver repair [63], and its deficiency impairs liver regeneration [64]. The concurrent downregulation of FGFR2b and FGFR4 in aged HFD-fed mice suggests a compromised ability of the liver to recover from metabolic stress, further exacerbating dysfunction. Furthermore, β-Klotho, a key coreceptor for FGF21 signaling [65], was significantly downregulated in HFD-fed young mice liver, indicating a reduction in hepatic FGF21 receptor sensitivity. β-Klotho is required for FGF21-mediated lipid and glucose metabolism, and its downregulation under metabolic stress is a hallmark of FGF21 resistance [66, 67]. The observed β-Klotho suppression may impair hepatic FGF21 responsiveness, reducing lipid oxidation and insulin sensitivity. Given that FGF21 exerts its effects through the FGFR1c/β-Klotho complex [65], the loss of β-Klotho expression may further contribute to the observed suppression of hepatic FGF21 expression, creating a vicious cycle of metabolic dysfunction.

HFD significantly reduced the expression of paracrine FGF1, FGF9, FGF16, and FGF17, and intracrine FGF11 and FGF13 in young mice kidney, suggesting that metabolic stress impairs renal protection of FGF signaling. Our recent study observed a significant reduction in the renal expression of FGF1 in patients and mice with diabetic nephropathy [68] and supplementation of recombinant FGF1 protein significantly suppressed renal inflammation, glomerular and tubular damage, and renal dysfunction in type 2 diabetic mice [68, 69], validating a renal protection of FGF1 under metabolic stress conditions. In contrast, FGF2 increased in aged HFD-fed mice kidney, potentially as a compensatory mechanism against metabolic stress-induced renal injury. Similar trends have been reported in ischemia-reperfusion injury and acute kidney injury models, where exogenous FGF2 administration attenuated renal damage and protected against apoptosis of renal tubular epithelial cells [70, 71]. The roles of FGF9, FGF11, FGF13, FGF16, and FGF17 in kidney especially under aging and metabolic stress conditions remain largely unknown.

HFD also disrupted FGFR and coreceptor expression in kidney, with FGFR1c, FGFR3b, Klotho, and Lctl significantly downregulated in young HFD-fed mice, suggesting that early-life metabolic stress may suppress FGFs' protective signaling and accelerate renal dysfunction [72, 73]. Klotho, a protein that plays a crucial role in aging and metabolism [74], was reduced in aged mice kidney, which was consistent with its reduction with aging [75, 76] and kidney disease [31]. Similarly, FGFR1c and FGFR3b downregulation suggests a loss of FGF-mediated renal protection, consistent with endothelial FGFR1-deficient models [77], where loss of FGFR1 signaling disrupts kidney regeneration.

Most FGFs showed no significant differences across groups in skeletal muscle, except for FGFR1c and Klotho, both of which were significantly downregulated in young HFD-fed mice. FGFR1 is essential for FGF2-mediated proliferation of satellite cells [78], and its reduction may contribute to impaired myogenesis. Likewise, Klotho, which has been linked to muscle aging [79], may exacerbate muscle dysfunction when downregulated under metabolic stress.

FGF2, FGFR1c, FGFR2c, and FGFR4 were significantly downregulated following HFD feeding in gWAT, suggesting impaired adipocyte differentiation and metabolic homeostasis [80, 81]. There has been reported a similar decline of FGF2 expression in WAT of obese mice, indicating a potential negative correlation between FGF2 levels and adipose expansion [82]. Given its biphasic role in adipogenesis [82], where low levels promote fat formation and high levels inhibit it, HFD-induced FGF2 downregulation may be an adaptive response to excessive lipid accumulation, potentially altering WAT function and expansion. Interestingly, FGF13 was significantly upregulated in young HFD-fed mice. This is in agreement with evidence from single-cell transcriptomic analyses in humans and mice [83], suggesting that FGF13 upregulation may serve as an adaptive response to metabolic stress and lipid accumulation in WAT.

Unlike gWAT, most FGFs in sWAT remained unchanged under HFD, except for FGF1, which was markedly upregulated following HFD feeding. FGF1 is a key regulator of adipose tissue expansion [84] and insulin sensitivity [85], with FGF1 knockout mice displaying worsened glucose homeostasis and lipid accumulation under HFD conditions [85]. The increase in FGF1 expression in sWAT under HFD conditions may represent a compensatory response to metabolic stress, aiming to preserve adipose tissue function.

HFD generally increased FGF expression in BAT, with FGF9 showing particularly significant upregulation. It has been demonstrated that FGF9 activates the ERK1/2 signaling pathway via FGFR3, leading to UCP1 upregulation and enhancement of BAT thermogenesis [86]. Additionally, FGF9 expressions could be induced by thermogenic stimuli such as cold exposure and exercise, and loss of FGF9 in BAT impairs thermoregulation and reduces BAT thermogenic capacity [86]. The observed FGF9 upregulation in HFD-fed BAT may therefore represent a compensatory attempt to enhance BAT thermogenesis and energy expenditure, counteracting HFD-induced lipid accumulation. Further research is needed to clarify the specific regulatory mechanisms underlying FGF9 induction in BAT under HFD conditions. As an essential coreceptor for FGF21-mediated metabolic regulation [66, 87], β-Klotho reduction in aged HFD-fed mice suggests that aging and metabolic stress might diminish BAT responsiveness to endocrine FGF21, potentially compromising thermogenesis and energy expenditure [88].

### Limitations and Future Directions

A limitation of our study is its observational and cross-sectional design, which restricts our ability to draw causal inferences. Future studies incorporating interventions such as administration of recombinant FGFs or targeted gene manipulation (ie, knockdown or overexpression) will be necessary to confirm the functional implications of the observed changes in FGF signaling pathways. Additionally, our study was conducted exclusively in male mice, representing another limitation given the well-established sexual dimorphism in metabolism, adipose tissue biology, and FGF signaling pathways. It is conceivable that female mice might exhibit different responses in FGF regulation under metabolic stress and aging conditions. Future research should incorporate both sexes to fully elucidate sex-specific dynamics in FGF signaling and associated metabolic dysfunction.

## Conclusion

Our study provides a comprehensive analysis of tissue-specific alterations in FGF and FGFR expressions in response to metabolic stress induced by HFD and aging. We identified distinct patterns of FGF regulation across major metabolic tissues, highlighting potential roles in cardiac remodeling, hepatic metabolism, renal function, skeletal muscle maintenance, and adipose tissue homeostasis. Notably, the observed downregulation of key FGFs and receptors, including FGF1, FGF2, FGFR1c, and Klotho, under metabolic stress suggests compromised metabolic adaptability and accelerated organ aging. Conversely, the upregulation of FGF1 and FGF9 in BAT likely represents compensatory mechanisms aimed at preserving thermogenesis and energy balance. Collectively, these findings underscore the critical importance of FGF signaling in maintaining metabolic health and provide a foundational framework for future physiological and mechanistic research. Prospective studies employing targeted genetic or pharmacological interventions will be essential to validate these observational findings functionally, clarify underlying causal mechanisms, and establish a robust basis for investigating metabolic dysregulation associated with obesity and aging.

## Data Availability

Original data generated and analyzed during this study are included in this published article.

## Abbreviations

BAT: brown adipose tissue
FGF: fibroblast growth factor
FGFBP: FGF binding protein
FGFR: FGF receptor
HFD: high-fat diet
IPGTT/IPITT: intraperitoneal glucose/insulin tolerance test
ND: normal diet
qPCR: quantitative polymerase chain reaction
WAT: white adipose tissue
